# El interés del desinterés en la epidemiología de servicios y sistemas de salud

**DOI:** 10.18294/SC.2023.4365

**Published:** 2023-03-09

**Authors:** Hugo Spinelli

**Affiliations:** 1 Doctor en Salud Colectiva. Director del Instituto de Salud Colectiva, Universidad Nacional de Lanús, Buenos Aires, Argentina. hugospinelli09@gmail.com Universidad Nacional de Lanús Universidad Nacional de Lanús Instituto de Salud Colectiva Buenos Aires Argentina hugospinelli09@gmail.com

**Keywords:** Epidemiología, Toma de Decisiones, Práctica Profesional, Medicamentos, Tecnología Biomédica, Enfermedad Iatrogénica, Administración en Salud, Epidemiology, Decision Making, Professional Practice, Drugs, Biomedical Technology

## Abstract

Este trabajo se propone problematizar, discutir y publicizar la autoridad cultural de la medicina científica, desde una dimensión política, y la implementación de la epidemiología de los servicios y sistemas de salud, desde una dimensión técnica. A partir de los conceptos de *interés del desinterés*, de Pierre Bourdieu, y de *autoridad cultural de los problemas públicos* de Joseph Gusfield se analiza ¿por qué la información epidemiológica es tan poco utilizada para la evaluación y monitoreo de las prácticas clínicas, poblacionales, institucionales y territoriales?, ¿por qué domina una cultura de toma de decisiones sin información epidemiológica? Desde este marco conceptual, se aborda un cuerpo documental que permite recuperar la débil cientificidad que sustentaron o sustentan algunas prácticas del campo de la salud en diferentes momentos históricos, organizado en tres ejes temáticos: la práctica profesional asistencial, los medicamentos y las tecnologías biomédicas.

## Abstract (en)

This article intends to critique of the cultural authority of the medical sciences, opening up a discussion on its publicization from a political standpoint. At the same time, from a more technical standpoint, it proposes the implementation of an epidemiology of health systems and services. Based on Pierre Bourdieu’s concept of *interest in disinterestedness* and Joseph Gusfield’s notion of the *cultural authority of public problems*, it analyzes why epidemiological information is so rarely used in the evaluation and monitoring of clinical, population, institutional, and territorial practices. That is, why does the dominant culture of decision making eschew epidemiological information? Within this conceptual framework, a body of documental evidence is analyzed that sheds light on the weak scientific basis that upholds or that has underpinned certain practices in the health field at different historical moments. The discussion is organized around three major themes: assistentialist professional practice, medication, and biomedical technologies.

## INTRODUCCIÓN

*Creer en la medicina sería el colmo de la locura, si el no hacerlo no fuera una locura todavía mayor, pues de la abundancia de errores, con el tiempo han surgido muchas verdades.* *Marcel Proust*^1^

El campo de la salud^2^ se sustenta en la doxa epistémica de la vieja ciencia aún hegemónica, que asocia lo real a lo racional, entendiendo que lo ideal se traslada mecánicamente al mundo real. Esta premisa niega que el mundo real es relacional, y que en lo relacional hay intereses específicos, incluso el interés del desinterés. Esa doxa epistémica facilita la idea de un mundo racional que se retroalimenta en la fe tecnocrática, y que ha llevado a la deshumanización de la ciencia, ocultando los intereses existentes detrás de las racionalidades técnicas^2^^,^^3^^,^^4^^,^^5^^,^^6^^,^^7^. Todo ello viene conduciendo, desde hace décadas, a los sistemas de salud a nivel mundial, a una crisis de graves consecuencias sociales. 

Frente a esa realidad, postulamos a la epidemiología de los servicios y sistemas de salud, como la propuesta técnica más pertinente en términos de eficacia, eficiencia y efectividad para analizar y monitorear los diferentes eventos y acciones que conforman los procesos de salud-enfermedad-atención-cuidado en el campo de la salud. El devenir de la medicina científica se ha ido transformado en función de una lógica de mercado que se demuestra insaciable frente a la demanda de los recursos económicos, y con escasa o nula regulación por parte de los Estados.

Dos preguntas conducen este trabajo: ¿por qué la información epidemiológica es tan poco utilizada para la evaluación y monitoreo de las prácticas clínicas, poblacionales, institucionales y territoriales?, ¿por qué en el campo sociosanitario domina una cultura de toma de decisiones sin información epidemiológica tanto a nivel de la formulación de las políticas, de la gestión, como de la práctica clínica?^2^^,^^8^^,8,^^10^.

La discusión que llevaremos adelante es que la implementación de la epidemiología de los servicios y sistemas de salud transparentaría prácticas que afectan a la salud de las personas y a la economía de los Estados, y al amenazar intereses específicos sustentados en prácticas innecesarias, o actos de corrupción, recibe como respuesta el desinterés^3^^,^^4^^,^^5^^,^^11^, el cual ejerce sin dificultades dada la autoridad cultural que la medicina científica construyó frente al problema público de los procesos de salud-enfermedad-atención-cuidado^12^, lo cual representa un problema de dimensiones políticas y no técnicas.

### Historias recientes

Entre el 7 y el 10 de noviembre de 1983, la Organización Panamericana de la Salud (OPS) realizó en Buenos Aires, Argentina, en la Academia Nacional de Medicina en tiempos de dictadura militar^13^, el seminario “Usos y perspectivas de la epidemiología”. Allí, personas de las áreas sanitarias, administración, planificación y epidemiología, de distintos países de las Américas, expusieron sobre la necesidad de impulsar la epidemiología para acciones de evaluación, planificación e investigación. En esa reunión Kerr White describió la epidemiología como “la aplicación del método científico al estudio de la utilización, efectividad, administración, organización, financiación y eficiencia de los servicios de salud que se dan directamente a las personas”^14^. Al año siguiente del encuentro, la OPS publicó el informe final del seminario, del cual extractamos los siguientes fragmentos:

En los servicios de salud, el uso de la epidemiología se ha concentrado en el desarrollo de sistemas de vigilancia orientados casi exclusivamente a detectar situaciones anormales, para permitir una intervención rápida de control, especialmente para algunas enfermedades transmisibles. En muchos países, estos sistemas se han convertido en mecanismos pasivos de notificación de casos caracterizados por la recolección de datos en los niveles periféricos y su consiguiente recopilación en los niveles centrales. En general, estos datos solamente cubren parte de la población (usualmente la atendida por los servicios públicos); su calidad es limitada por deficiencias en los servicios de diagnóstico, y no son motivo de análisis en los niveles de prestación de servicios. Esta situación se ve agravada por la multiplicidad de formularios empleados para la notificación de casos de enfermedades, cuyo control, normalización y supervisión dependen de programas distintos e independientes entre sí. Aún en las pocas circunstancias en que estos datos son analizados localmente, la información obtenida no genera acciones inmediatas […] En los niveles centrales, los datos así obtenidos, además de ser poco confiables, carecen de oportunidad. Gran parte de la información divulgada está circunscrita a tablas estadísticas con escaso o ningún análisis. La etapa del diagnóstico de salud generalmente se limita a tasas o indicadores nacionales que no revelan las variaciones geográficas y sociales que existen o puedan existir en cada país […] La experiencia obtenida durante el decenio de 1970 mostró que la incorporación de tecnologías avanzadas no generaba, en la mayoría de los casos, los beneficios observados en los países en donde esta tecnología se había perfeccionado. La aceptación y adquisición de tecnología -más que de conocimiento- independientemente de las posibilidades reales de su uso, ha sido el mecanismo más generalizado de incorporación del desarrollo tecnológico en la Región y en muchos casos ha resultado inadecuado^14^.

Cuatro años después de esa reunión, se realizó la XIV Conferencia de la Asociación Latinoamericana y del Caribe de Educación en Salud Pública (ALAESP), en Taxco México, bajo el título “La formación en epidemiología para el desarrollo de los servicios de salud”^15^. En la sesión inaugural, Ronald St. John, coordinador en la OPS del Programa de Análisis de la Situación de Salud y sus Tendencias, recuperó los temas tratados en la reunión de Buenos Aires, y formuló tres preguntas: “¿Dónde estamos en materia de capacitación en epidemiología?, ¿dónde quisiéramos estar de aquí a cinco años y en el año 2000?, ¿qué y cómo debemos hacer para llegar allí?”^15^

Luego de esas preguntas y para cerrar su exposición afirmó:

Si durante los próximos días el énfasis de las discusiones se centra en la importancia de la epidemiología o de los epidemiólogos, esta no será sino otra reunión más entre las muchas que ya se han realizado […] Para que este encuentro cumpla su propósito, es preciso que los debates resulten en sugerencias y recomendaciones acerca de estrategias y acciones concretas de capacitación que conduzcan a hacer realidad la incorporación del pensamiento y la práctica epidemiológica en todos los ámbitos y niveles de los servicios de salud. Este es nuestro desafío durante los próximos días^15^

En el año 1988, la OPS publicó *El desafío de la epidemiología*^16^, obra coordinada por Carol Buck, Álvaro Llopis, Enrique Nájera y Milton Terris. En la parte IV de esa publicación, titulada “Servicios de salud y políticas de salud” Terris argumenta:

Toda la historia de la epidemiología ha sido una crónica de los estudios etiológicos […] la epidemiología debe atenerse a la evaluación de los resultados, al *efecto* que los servicios de salud ejercen en la salud […] En América Latina, por ejemplo, quieren utilizar la epidemiología con objeto de evaluar los servicios de salud disponibles, incluidos los de atención médica […] Debemos reclamar un papel para la epidemiología en la investigación de los servicios de salud y hacer que ese papel sea prioritario. Los economistas se han apoderado del campo y tenemos que recuperarlo de ellos*.*^16^

En tanto Nájera sostiene:

…la epidemiología tiene tres usos principales: la planificación de los servicios de salud, la organización y administración de esos servicios, y la investigación sobre causalidad y sobre nuevos métodos de estudio. Desde los decenios de 1950 o 1960 todo el mundo ha convenido en que la epidemiología es la ciencia básica para planificar, organizar y evaluar los servicios de salud, pero, con excepción de la evaluación de los programas verticales o de determinado tipo de atención médica, la epidemiología nunca se ha utilizado en realidad para eso […] La investigación de los servicios de salud ha adquirido cada vez más importancia, en la organización de los mismos. Pero sin epidemiología, la investigación de los servicios de salud es simplemente de tipo administrativo. Se concentra en que haya mejor administración, mejores técnicas de gestión.^16^

A cuatro décadas de la reunión en la Academia Nacional de Medicina y 25 años de la publicación de El desafío de la epidemiología^16^ es evidente lo poco que se ha avanzado en la epidemiología de los servicios y sistemas de salud. Lejos se está de la esperanzadora concepción de la epidemiología que formuló Kerr White en la reunión de Buenos Aires “la epidemiología debería aceptar el hecho de que es una ciencia social además de biológica y que, como toda actividad social, debe servir a la sociedad”^14^.

### Cuestión social, problemas sociales y problemas públicos

A partir del siglo XIX, los primeros textos sociológicos en los EEUU consideraron los problemas sociales como herederos de la cuestión social de la época, caracterizada por la desorganización familiar, la explosión urbana, la delincuencia juvenil, la cuestión racial, el trabajo infantil, el desarrollo del vicio, la asimilación de los inmigrantes, o la criminalidad de los pobres^17^.

Robert Ezra Park, fundador de la Escuela de Sociología de Chicago entendía a los problemas sociales íntimamente ligados a procesos históricos:

*…la historia natural de los problemas sociales no difería de la historia del “proceso político” que lo engendra […] el problema social es el resultado de un proceso histórico en que muchos individuos participaron sin prever cuál sería el desenlace de sus esfuerzos.*^17^

Para Gusfield, no todos los problemas sociales llegan a ser problemas públicos^12^:

*Los problemas sociales son lo que la gente piensa que son, y si las condiciones no están definidas como problemas sociales por la gente implicada, entonces no hay problema para esa gente, aunque puede haberlo para observadores o para científicos […] El proceso de surgimiento, configuración, estabilización e institucionalización de los problemas públicos puede pensarse como resultado de correlaciones de fuerza o de conflictos de interés que se ponen en juego en diferentes sectores: las altas esferas de la acción pública, las asambleas legislativas y las agencias administrativas, los laboratorios científicos y las organizaciones asociativas, la redacción de los periódicos u, hoy en día, los foros y las plataformas de internet.*^17^

Gusfield prefiere llamarlos problemas públicos y no sociales, porque entiende que hay problemas sociales que pueden ser privados^12^. Para fundamentar la posición de Gusfield, Cefaï recurre al pragmatismo de John Dewey, quien en 1927 escribía en EEUU:

*Nuestro punto de partida es, por tanto, el hecho de que los actos humanos tienen consecuencias en las demás personas, y algunas de esas consecuencias son percibidas, y su percepción lleva a un esfuerzo ulterior para controlar la acción de manera de asegurar algunas consecuencias y evitar otras. Esta indicación, nos lleva a señalar que las consecuencias son de dos tipos: aquellas que afectan a las personas directamente implicadas en una transacción y aquellas que afectan a otras, más allá de las que sí están inmediatamente afectadas. En esta diferenciación, encontramos el germen de la distinción entre lo privado y lo público. Cuando se reconocen consecuencias indirectas y se hace un esfuerzo por reglamentarlas, comienza a existir algo con algunas características de Estado. Cuando las consecuencias de una acción están principalmente limitadas (o cuando se cree que están limitadas) a personas directamente implicadas, la transacción es privada.*^17^

Gusfield entiende los problemas públicos como construcciones sociales e históricas que cambian en el tiempo, relacionadas a cuestiones estructurales y culturales. En síntesis, reconoce que el campo de las experiencias genera dinámicas en las que ocurren transacciones entre personas, grupos y sus entornos, y cuando se producen quiebres surgen los problemas públicos que interpelan las formas organizativas y las culturas, dando lugar a las denuncias y los intentos de negar u ocultar aquello que se tornó visible. Frente a los problemas públicos se construyen autoridades culturales que pasan a ser los propietarios del problema, contando a su favor que su competencia está fuera de toda sospecha^12^^,^^17^.

La historia de los problemas sociales no es lineal, pueden ocurrir saltos, interrumpirse procesos, pasar por momentos de transición y de traducción, con puntos de bifurcación y bucles de retroalimentación, lo cual señala la incertidumbre que constituye su devenir, que anula la idea de modelos únicos para sus abordajes. Los problemas sociales están preconfigurados a la espera de expresiones y configuraciones narrativas^17^. Es muy raro que los problemas públicos surjan de manera clara, ya que son construcciones socioculturales que muchas veces no se las quiere reconocer^12^^,^^18^. Por ello, en general, los problemas públicos se basan en argumentos sesgados, construidos muchas veces con información científica de dudosa calidad^19^. Tampoco todos los problemas sociales llegan a ser problemas públicos porque no se problematizan, o porque hay intereses específicos que no lo permiten y, por lo tanto, no se publicizan, es decir no se los hace públicos^17^^,^^20^^,^^21^.

Los problemas públicos remiten a campos de experiencias^17^. Gusfield los define como tales cuando hay una autoridad cultural que se presenta como propietaria del problema, la cual se legitima al poder responder las preguntas ¿cómo es posible?, ¿qué hacer? y ¿quién lo hace? Las respuestas a estas preguntas se formulan, en general, desde una lógica causal, definiendo la tarea, y asignando los responsables^12^. La autoridad es en cierta parte ilusoria porque sus fuentes son ilusiones producto de una mezcla entre lo instrumental y lo simbólico, donde no solo hay razón, conocimientos y método científico, sino también irracionalidades, conflictos e intereses^12^.

Para escapar de la ilusión que genera la autoridad cultural, los problemas públicos deben ser interpretados desde lo sociocultural, ya que están constituidos por áreas de conflictos entre diferentes actores que representan intereses específicos y, a veces, antagónicos. Son actores que disputan la autoridad sobre el problema o su desatención^12^^,^^17^.

Para Gusfield la autoridad cultural sostiene su posición con recursos propios del arte dramatúrgico, ejerciéndolo a través de acciones simbólicas y comunicacionales, apelando a la retórica o a la narrativa, con oratorias que expresan dramas, relatos, o argumentos, con los cuales se busca implicar a los “espectadores” con un guion, para convencerlos o persuadirlos. En el caso de la medicina, el ejercicio del arte dramatúrgico se acompaña de ciertos ritos, como el guardapolvo blanco, y de mitos, como la historia abnegada de los grandes médicos^12^^,^^17^.

La ciencia hegemónica jerarquizó lo experimental y no el lenguaje, ya que entendía que de esa manera se producían datos objetivos que permitían la descripción y el análisis, y así poder construir verdades universales. Por ello, el lenguaje fue reprimido y todo atisbo literario fue considerado no científico. El lenguaje se redujo a una mera herramienta neutra, útil para describir cuestiones consideradas objetivas. Sin embargo la medicina científica siempre necesitó y necesita persuadir y convencer, y para ello se vio obligada a recurrir a la argumentación, buscando validarse en auditorios universales, para así legitimar su autoridad cultural a través de una discusión argumentativa^12^^,^^22^.

La autoridad cultural tiene, en general, un detrás de escena, un fin privado que puede ser económico o de control social y que, en función de esos fines, despliega actos instrumentales, acciones ceremoniales, o ambos^12^. Y así como para el ejercicio de la autoridad científica recurre a explicaciones, argumentos y creencias, para el ejercicio de la responsabilidad política recurre a programas y a cuestiones jurídicas^12^. Las ideas anteriores son contrarias a como la ciencia es relatada y enseñada. En esos relatos los científicos tienen destinado el rol de hacer descubrimientos que los técnicos aplicarán para resolver problemas de la sociedad. Esas ideas constituyeron un imaginario social que permitió que los problemas públicos queden en manos de los técnicos que vieron así facilitado el camino para constituirse en autoridades culturales de los problemas públicos, autoridad que ejercen a través de la argumentación. Esta última, carecía de cientificidad y debía ser anulada según el discurso de la ciencia hegemónica, pero no pudo hacerlo ya que la necesitó, no por cuestiones racionales, sino por cuestiones socioculturales^12^. Así, la necesidad de acudir a la retórica y a la argumentación evidencia el ejercicio de la medicina como autoridad cultural. Todo este proceso construyó un gran aparato ideológico basado en la argumentación científica.

Esa autoridad cultural puede recurrir, si es necesario, a formas de control social, excluyendo la posibilidad de conflicto y divergencia, ya que no hay alternativas a su relato que se basa en la presunta legitimidad de sus juicios morales y cognitivos, compartidos por la sociedad y socializados por los medios de comunicación social. Todo ello legitima que el problema solo puede ser modificado por la autoridad cultural^12^^,^^17^.

La ciencia, las declaraciones de los científicos, sus publicaciones, los programas específicos y las tecnologías respaldan la autoridad cultural de la medicina, otorgándole un matiz de neutralidad que retroalimentan los medios de comunicación social; proceso por el cual se validan los argumentos de la medicina científica frente a los problemas sociales y los problemas públicos.

A los problemas públicos, en general, se los subordina a una cientificidad que se nuestra ciega ante sus dimensiones sociales y, en general, se busca responsabilizar o culpar a las propias víctimas del problema. Para ello, se montan modelos explicativos de lógica causa-efecto, complejos modelos matemáticos o modelos sistémicos con sus cajas negras, evitando que se produzca la elucidación del problema por parte de los conjuntos sociales. En esos casos, no importa que la respuesta no sea científicamente válida, se busca que se comprenda y acepte por legos, y si los argumentos se basan en datos, rara vez se explicita quien los toma, qué se selecciona, y qué no se selecciona^10^^,^^12^.

El problema social de los procesos de salud-enfermedad-atención-cuidado se transformó en un problema público, por el cual la medicina científica, en el marco de un proceso político, se constituyó en su autoridad cultural a partir del siglo XX. Esta idea la desarrollaremos en el apartado de las ideas a los hechos, en el que recuperaremos evidencias en revistas científicas que se respaldan más en el ejercicio de la autoridad cultural de la medicina científica que en fundamentos científicos^2^.

### Un juego sin árbitros

Existe un ideal en la administración y gestión empresarial sustentado en que debe haber una coherencia entre la producción, los ingresos económicos y el gasto. Esa lógica en el campo de la salud se convierte en un laberinto insondable.

Pensemos por un momento las caras y expresiones de los directivos de una empresa al leer el párrafo anterior, o los párrafos que recuperamos del informe del seminario que organizó la OPS en Buenos Aires en 1983^15^. ¿Cómo les explicamos esa dinámica?, ¿qué dirían si además tuvieran en cuenta que un alto porcentaje de sus integrantes son egresados universitarios? Será muy difícil que puedan entenderla. ¿Los trabajadores de la salud la entienden?, ¿o se resignan?, ¿cuál es el costo que pagan por esa resignación?

Para entender la dinámica del campo de la salud es necesario interpretar el trabajo en salud desde su carácter ontológico, su esencia, que es artesanal y por ello está fuera de la teoría general de la administración formulada a fines del siglo XIX por Taylor y Fayol, y formulada para superar los modelos artesanales^23^. Esa teoría organizativa fue muy “exitosa” en el mundo industrial y comercial, pero acumula una cadena de fracasos en el campo de la salud, sobre todo si es pensada en términos de la salud de las personas y no en tasas de ganancia de los propietarios de las instituciones. Esos fracasos en el campo de la salud, se deben, como ya hemos señalado en otro trabajo^2^, a que la dinámica de la teoría general de la administración está pensada para interacciones entre un sujeto y un objeto, lo cual permite la acción racional orientada a fines, pero es inadecuada en procesos relacionales donde el trabajo se da por la interacción entre sujetos mediados por el lenguaje. Por ello reconocemos y jerarquizamos el carácter artesanal del trabajo en salud, en el que confluyen elementos de la ciencia, del arte y del juego, y donde muchas veces se decide “qué hacer” en el acto, y sin certezas de los resultados. Es un hacer basado en conocimientos, experiencias y conjeturas^24^. Por ello, es un trabajo que, en general, solo es posible de supervisar estando en la situación o con posterioridad a la acción.

Esta descripción evidencia los niveles de libertad del trabajador del campo de la salud, libertad que ni los anarquistas soñaron. Esa libertad produce una mutación de la configuración clásica de la burocracia mecánica (la pirámide conformada por el *homo sapiens* y el *homo faber*), y de esa mutación surge la burocracia profesional (donde al *homo sapiens* y al *homo faber* se suma con un rol central el *homo ludens*)^2^^,^^25^. Defendemos radicalmente el trabajo artesanal en salud, y la importancia del *homo ludens*, que produce una dinámica institucional que se ubica en las antípodas de la teoría general de la administración. También debemos reconocer que esas características del juego son las que lo hacen muy difícil de evaluar.

### El interés del desinterés

La doxa de un campo configura un conjunto de creencias en lo que está en juego, y en el orden del juego. La doxa es una construcción que deviene una verdad irrefutable, instalada en el sentido común de quienes juegan el juego. Todo ello configura, para Bourdieu, una tradición basada en jugar y respetar las reglas del juego, y aunque se las transgreda, se las respeta^3^. A su vez, esa verdad irrefutable, se sustenta en una doxa epistémica contraria a la lógica de los campos sociales, sustentada en la idea de un mundo racional, que habilita la fe tecnocrática, lo que llevó a la deshumanización de la ciencia, a la vez que ocultó los intereses existentes detrás de las racionalidades técnicas^2^^,^^3^^,^^4^^,^^5^^,^^6^.

En todo campo, siempre hay intereses y desintereses. El interés es una disposición individual o colectiva, es la ausencia de indiferencia y, por lo tanto, se opone al desinterés, pero siempre hay intereses y desintereses^3^.

Cuando no hay interés, puede deberse a que el agente no ve el juego, no lo atrapa y por ello no invierte, es decir no hay *illusio* en el juego. Esa situación es diferente a cuando el juego que se propone es contrario a los intereses del jugador, entonces puede decidir participar desde el desinterés, que es una condición para poder seguir obteniendo los beneficios que constituyen su *illusio*^4^. El interés del desinterés será más poderoso en el juego si quienes lo expresan son los dueños del problema, los que tienen la autoridad cultural sobre el tema^3^^,^^12^^,^^17^.

El juego en el campo de la salud no se centra solo en el cuidado y la atención de las personas y las comunidades, hay jugadores que no priorizan esos valores, como también hay quienes, en función de sus posiciones en el campo, pueden cambian sus acciones y pasar a hacer y decir lo que criticaron en otro momento, el mejor ejemplo son las prácticas de la *American Medical Association* (AMA), que analizaremos más adelante en este artículo^3^^,^^5^^,^^11^^,^^26^.

En la conformación de la doxa del campo de la salud no hay espacio para la epidemiología de los servicios y sistemas de salud, porque su propósito de darle transparencia a las acciones es contrario a los fuertes intereses específicos de las autoridades culturales del problema, que saben que de implementarse, desnudaría acciones que van en contra de la salud de las personas, que incrementan el gasto en salud y se verían afectados sus intereses. Los actores que representan esos intereses específicos no confrontan con la propuesta de la epidemiología de los servicios y sistemas de salud, ya que hacerlo los obligaría a “mostrar sus cartas”. Ellos juegan fieles a su *collusio,* entendida como inteligencia en perjuicio de terceros, por ello deciden ignorar la epidemiología de los servicios y sistemas de salud, y responder con el desinterés. Sin el esfuerzo del cálculo sobre las acciones y sin explicitar sus intereses, alcanza los objetivos de su juego. Ese desinterés que demuestran es parte de las condiciones de su éxito y expresión de que son buenos herederos y reproductores de la doxa del campo^3^^,^^5^^,^^6^^,^^11^.

#### La construcción de la medicina científica como autoridad cultural del problema público de la enfermedad

La fuerte relación entre condiciones de vida y morbimortalidad en los conjuntos sociales se hizo evidente con el surgimiento de la revolución industrial, lo cual llevó a que las enfermedades aparecieran como una cuestión social.

Antes del siglo XX, las enfermedades se enfrentaban básicamente con acciones sanitarias concentradas en la provisión de agua potable, saneamiento ambiental, y combate tanto a las epidemias como a las prácticas médicas muy poco sistematizadas, ejercidas por médicos con títulos dudosos, barberos, charlatanes y curanderos^9^^,^^26^.

En la segunda mitad del siglo XIX, los descubrimientos de Pasteur inician la conformación de la medicina científica. Es recién allí cuando la salud pasa de ser una cuestión social a convertirse en un problema público, ante el cual la medicina científica va construyendo una legitimidad cultural tal, que la transforma en la autoridad cultural del problema público que representan los procesos de salud-enfermedad-atención-cuidado^12^.

Antes de los descubrimientos de Pasteur, el pensamiento epidemiológico se limitaba a describir diferentes formas de morir y enfermar relacionadas con variables como edad, sexo, lugar, trabajo, guerras o epidemias^16^^,^^27^^,^^28^^,^^29^^,^^30^. Pero en la segunda mitad del siglo XIX, la revolución pasteuriana, al basarse en la teoría del germen y del contagio, desencadena un proceso de legitimidad de la medicina científica que conduce a una fuerte jerarquización social del hospital, el cual toma el modelo institucional de la fábrica, a pesar que el trabajo en salud es por carácter ontológico artesanal y no industrial^2^^,^^26^.

A partir de los descubrimientos de Pasteur, la medicina científica se apropia del problema público de la salud, en disputas con la iglesia, los propios médicos y los gobiernos. En la legitimación de la medicina científica, el positivismo tuvo un rol muy importante, al basar la investigación biomédica en el método experimental como el único método científico válido, que debía realizarse en los laboratorios del hospital, de manera de asegurar que las variables sociales no sesguen los resultados.

La medicina científica comenzó a enseñarse en el siglo XIX en las universidades alemanas. Allí se formaban médicos imbuidos en los principios de la nueva medicina basada en los principios pasteurianos, y hacia allí viajaron los jóvenes de EEUU a estudiarla. Fueron ellos quienes al volver a EEUU denunciarían las irregularidades en la extensión de los títulos de médicos, la falta de controles de los requisitos que debían cumplir los estudiantes para egresar, y la venta de medicamentos carentes de fundamentos científicos. Todo ello llevará al Informe Flexner^26^^,^^31^^,^^32^.

A partir de la década de 1930, los descubrimientos y avances científicos en la clínica, los medicamentos y las tecnologías incrementaron la complejidad del campo de la salud a nivel mundial. Ello transformó a las instituciones de salud, y subordinó los procesos de salud-enfermedad-atención-cuidado bajo la autoridad cultural de la medicina científica.

Si bien existen publicaciones que señalan críticamente las prácticas médicas científicas por su sobreutilización durante la primera mitad del siglo XX, es sobre todo a partir de la segunda mitad de ese siglo que las publicaciones críticas toman una dimensión insospechada, demostrando cómo diferentes procedimientos y terapéuticas carecen de evidencias científicas para ser realizadas. Esa dinámica del campo de la salud acompañó el proyecto de la *Big Science* impulsado por EEUU luego de la Segunda Guerra Mundial^33^. Así, la sobreutilización de servicios asistenciales y diagnósticos, la creciente sobreindicación de medicamentos^34^, tecnologías^9^, y la fuerte medicalización de la sociedad produjo gastos desmedidos que no guardaron relación con los resultados en salud^2^. A inicios del siglo XX en EEUU, la tasa de ganancia se concentraba en los profesionales, sobre todo médicos, pero a partir de la década de 1970 comienzan a resignarla frente a la fuerte presencia del complejo médico industrial^35^, produciéndose una brecha financiera y una puja distributiva que se extendió por la mayoría de los países y regiones^9^^,^^24^^,^^36^

En la década de 1970, David Mechanic, prestigioso sociólogo dedicado al estudio de la salud, afirmaba sobre los médicos:

*Los cerca de 400.000 médicos que hay en Estados Unidos disfrutan de una desproporcionada influencia social y política. Ejercen un vasto control sobre la organización y provisión de servicios médicos y medios de trabajo de casi todos los demás profesionales de la salud, también es desproporcionada su influencia sobre la política sanitaria y social, por su alta posición social y su red de grupos de interés que sirven a propósitos políticos más allá de los profesionales.*^37^

Starr señala que, a partir de la década de 1970, en EEUU se produce “el fin de un mandato”, en alusión a la reducción del poder de la Asociación Médica Americana (AMA), por la pérdida de confianza de la ciudadanía en las instituciones de salud. Esto cuestiona una de las premisas que había dominado hasta entonces la formulación de las políticas de salud: “se necesitaba más atención médica, y que los profesionales y las instituciones estaban perfectamente capacitadas para organizar esas prestaciones”^26^. A esas críticas se sumaron los economistas argumentando que las inversiones sociales crecientes no eran efectivas en relación con los costos que demandaban. Las críticas a la atención médica fueron parte de un movimiento que cuestionó también los servicios sociales^26^.

*En la década de 1970 este mandato se acabó. Los problemas económicos y morales de la medicina desplazaron el progreso científico al centro mismo de la atención pública. Aumentos enormes en los costos coincidieron con dudas sobre los beneficios en las mejoras en la salud. Se invirtieron los supuestos dominantes sobre la necesidad de aumentar la atención médica, la necesidad pasaba por frenar el apetito aparentemente insaciable de recursos de la medicina científica.*^26^

La autoridad cultural de la medicina comenzó a mostrar señales de debilidad. Aunque de manera coincidente con las críticas, se produjo la disminución más importante del siglo XX en las tasas de mortalidad en EEUU, sobre todo en aquellas causas de muerte más sensibles al tratamiento médico, no así en las relacionadas con problemas sociales como las violencias. Para Starr esa caída en la mortalidad debería relacionarse también con que fueron años donde se realizaron controles sobre la contaminación ambiental y se mejoró la alimentación de la población con programas de ayuda a los pobres. Y sintetiza así ese período: “En un lapso muy breve, la medicina estadounidense pareció dar el salto de la escasez tenaz al exceso irrefrenable, sin haber conocido en ningún momento una suficiencia feliz”^26^.

En ese fin de mandato surge un nihilismo terapéutico que planteó que los medicamentos y terapias existentes eran inútiles. Ese movimiento se inició contra la psiquiatría y los hospitales psiquiátricos, y luego se extendió a otras especialidades. Ese nihilismo sobre la medicina científica se proyectó en el tiempo, lo cual se evidenció en la pandemia de Covid-19 a través de los movimientos antivacunas en diferentes países^38^^,^^39^. Los límites cada vez más difusos entre profesión y negocios comenzaron a socavar los cimientos de la autoridad cultural de la medicina.

A partir de las últimas décadas del siglo XX las publicaciones críticas sobre las prácticas médicas, los medicamentos y las tecnologías se volvieron cada vez más frecuentes. Escritas por académicos y profesionales del campo de la salud^9^^,^^19^^,^^34^^,^^40^^,^^41^^,^^42^, académicos y periodistas en trabajos conjuntos^41^ o por periodistas especializados en temas de salud^44^^,^^45^^,^^46^^,^^47^^,^^48^^,^^49^^,^^50^.

## ACERCA DE LA INVESTIGACIÓN

Para este trabajo se compilaron publicaciones que señalan la débil cientificidad que sustentaron o sustentan algunas prácticas del campo de la salud en diferentes momentos históricos y se organizaron en tres ejes temáticos: la práctica profesional asistencial, los medicamentos y las tecnologías biomédicas. Utilizamos como fuentes dos antologías publicadas por la OPS en 1988^16^ y 1992^51^, y una selección de artículos y libros. Esa débil cientificidad se sostiene en el tiempo y en diferentes países, producto de un proceso de racionalización de la medicina, que constituye una forma de dominio político, muy lejano de la idea de una razón técnica neutra e impoluta. Para Marcuse, el propio concepto de razón técnica es ideológico, en tanto se relaciona a un proyecto histórico social^7^. Las referencias tienen en común, en general, estar sustentadas más en su autoridad cultural sobre el problema público de la enfermedad que en sus bases científicas^12^.

En el caso de las citas anteriores a 1950, para constatar la persistencia de esas prácticas, buscamos citas actuales en PubMed y SciELO. Aunque sabemos que es poco común la autodenuncia y que las corporaciones protegen y también amenazan^52^, entendemos que no encontrar publicaciones actuales, no significa que el problema haya sido superado, ni tampoco podemos afirmar que se continúen realizando.

## DE LAS IDEAS A LOS HECHOS

### La práctica asistencial: de lo humano a lo mensurable

El historiador de la medicina Johann Hermann Baas afirma que el sistema browniano impulsado por John Brown en la segunda mitad del siglo XVIII, al que clasifica como charlatanismo, había provocado más muertes de seres humanos que la suma de las ocurridas en la revolución francesa y las guerras napoleónicas^53^.

Las bases jurídicas de la responsabilidad médica surgen en Francia en 1825 y se conocen como el *Proceso Helie*. Es la historia de un médico que, ante un parto distócico con presentación de hombro y mano derecha en el trayecto vaginal, procede a su amputación y al darse la misma situación con el otro brazo, también lo amputa. El niño sobrevive y el caso es llevado a la justicia. El tribunal consultó a la Academia de Medicina de Francia, y con base en las opiniones de dos comisiones de esa Academia, el tribunal falló que no hubo intencionalidad y declaró al médico no responsable^54^^,^^55^.

A mediados del siglo XIX en una maternidad de Viena, previo a la revolución pasteuriana, el médico Ignaz Semmelweis analizó la fiebre puerperal preocupado por la mortalidad de las parturientas y postuló, como causa de la fiebre, la falta de aseo de manos en médicos y practicantes, antes y luego de revisar a las parturientas. Esa hipótesis ofendió a sus colegas y le valió la reprobación y consiguiente marginación, que lo llevó a terminar su vida internado en un psiquiátrico donde muere en 1865^16^^,^^25^. Buck considera el trabajo de Semmelweis como el primer estudio epidemiológico sobre iatrogenia médica^16^^,^^55^. La hipótesis de Semmelweis se confirmó con los descubrimientos de Pasteur quien, junto a Joseph Lister, fundamentaron la importancia del lavado de manos, que constituyeron las bases de la asepsia y antisepsia en cirugía, y redujeron con ello la mortalidad de las puérperas al 1%^27^. El lavado de manos sigue siendo la principal recomendación de los comités de infecciones de los hospitales ante las infecciones, no obstante todo el conocimiento producido, la principal causa de infección intrahospitalaria en la actualidad continúa siendo el no lavado de manos del personal^56^.

El cuerpo de la mujer fue y es objeto de prácticas innecesarias construidas desde lógicas patriarcales. Burnham describe por primera vez en 1853 que de 15 pacientes a las que se les realizó una histerectomía abdominal, solo tres sobrevivieron, lo que significa una tasa de mortalidad del 80%^16^.

En la década de 1930, se publicó en *New England Journal of Medicine* que, en Inglaterra y Gales, la incidencia de tonsilectomía -extracción de amígdalas- se había generalizado en niños provocando muertes, y que los más afectados eran niños de clases altas^51^^,^^57^. En EEUU, en la década de 1930, cada año 200 mil niños eran amigdalectomizados y esa cifra representaba un tercio de las operaciones realizadas con anestesia general. A pesar de esa evidencia científica, luego de la Segunda Guerra Mundial el procedimiento quirúrgico recuperó su fama, y en 1958 una investigación en EEUU publicada en *Journal of Pediatrics* señaló su persistencia e indicó como responsables de su impulso a los diarios y a la publicidad de los seguros médicos^51^^,^^55^^,^^58^. ¿El problema se mantiene vigente? En 2011, en EEUU, se realizaron 289.000 amigdalectomías anuales en menores de 15 años^59^Otro artículo del año 2006^60^ señala que, a pesar de la alta frecuencia de adenoidectomías y amigdalectomías, estas cirugías presentan escasas complicaciones perioperatorias. Los autores deberían haber aclarado que las escasas complicaciones no son sinónimo de que se las recomiende de manera generalizada. El mismo artículo se encuentra publicado en 2007, en la revista de *Otorrinolaringología de Cirugía, Cabeza y Cuello* en la sección “Revista de revistas”^61^.

En 1936, las observaciones de Fulton y Jacobsen sobre la función psíquica del lóbulo frontal en monos le permitieron, en 1936, a Antonio Egas Moniz practicar las primeras lobotomías y, en 1949, alcanzar el premio Nobel de Medicina, por su trabajo sobre el valor terapéutico de las leucotomías en las psicosis^55^. La lobotomía en las décadas de 1930 y 1940 tuvo amplia indicación para el tratamiento de problemas psicológicos o neurológicos, algunos de ellos hoy considerados menores pero que, sin embargo, recibían la indicación de recesión de un lóbulo cerebral, o parte de él. Las entidades nosológicas que recibían tales prescripciones eran las autoagresividades^62^, la esquizofrenia^63^, los desórdenes mentales^64^, o las psicopatías^65^^,^^66^. Las lobotomías continúan siendo objeto de publicaciones^67^.

A fines de la primera mitad del siglo XX, en Inglaterra, se describieron los efectos de la hospitalización en niños y niñas, al negarle a la familia, sobre todo a la madre, ser parte de la internación. Esa actitud ponía en evidencia la ignorancia de los profesionales sobre las dimensiones que hacen a la salud en la niñez y pertenecen al ámbito de los afectos y su sociabilidad^68^^,^^69^. En Argentina, esa situación fue descripta por Escardó y Giberti en 1964, quienes criticaron la medicalización de la infancia y afirmaron “la medicina está enferma de ciencia”^70^. La problemática de la hospitalización se sigue discutiendo y se buscan formas de monitorear tanto las reinternaciones como las estadías prolongadas^71^.

Paul Lembcke, profesor de la Escuela de Higiene y Salud Pública de la Johns Hopkins University, y pionero en la evaluación de la atención médica, publicó en el *American Journal of Public Health,* en 1952, un trabajo sobre apendicetomías primarias (llamadas así cuando el objetivo es extirpar el apéndice) y apendicetomías secundarias (cuando se la realiza preventivamente en el marco de otra operación). El estudio se realizó en una región de 860.000 personas al oeste del estado de Nueva York, las que eran atendidas gratuitamente por el Consejo de Hospitales Regionales de Rochester. Los resultados muestran que durante el año 1948 hubo un total de 3.280 apendicectomías primarias, lo cual representó una tasa bruta de 3,83 extirpaciones por mil habitantes, y un total de 1.372 apendicectomías secundarias, con una tasa bruta de 1,60 por mil habitantes. Al analizar la correlación entre ambos tipos de apendicectomías se encontró que la única explicación radicaba en el tamaño y complejidad del hospital, ya que cuando mayor era el hospital, mayor era la correlación^72^. En la actualidad el tema se sigue discutiendo^73^^,^^74^.

En 1953, Doyle publica en el *Journal of the American Medical Association* el trabajo sobre histerectomías innecesarias^75^. Allí analiza 6.248 casos en California y encuentra que, en 144 pacientes, la intervención estaba contraindicada; en 182, la intervención se consideró exagerada y, en 788 casos, no se constató ningún otro signo al examen físico más que el dolor abdominal, sin evidencias microscópicas en la anatomía patológica^51^. En la actualidad se siguen realizando histerectomías en patologías graves que la bibliografía considera innecesarias en determinadas condiciones^76^. También hay referencias actuales en Cuba^77^ y en México^78^ que evidencian que el cuerpo de la mujer sigue siendo un objeto de aprendizaje y de prácticas que no respetan sus derechos.

En 1963, se publica en *Medical Care* un estudio realizado en Londres sobre la letalidad en hospitales entre 1956 y 1957, que señala que la letalidad era un 40% menor para ciertas afecciones en los hospitales escuelas que realizaban prácticas de enseñanza, en comparación con aquellos hospitales que no realizaban prácticas de enseñanza^51^.

En 1969, Charles Lewis publica en *New England Journal of Medicine* un análisis de los registros de la *Kansas Blue Cross Association*, en el que muestra que, en algunas regiones, las tasas de intervenciones quirúrgicas eran tres a cuatro veces mayores que las encontradas en otras regiones, y las intervenciones más frecuentes correspondían a amigdalectomías, apendicectomías y herniorrafías. La única asociación que se encontró fue una mayor cantidad de camas hospitalarias y de cirujanos como las variables más representativas^51^.

En 1973, Eugene Vayda publica en *New England Journal of Medicine* un estudio que compara las tasas de cirugía de Canadá (año 1968), Gales (año 1967) e Inglaterra (año 1967), y muestra que las tasas de cirugía en Canadá fueron 1,8 veces superiores en los hombres y 1,6 veces en las mujeres, que en Inglaterra y Gales; y que la tasa estandarizada por edad y sexo de tonsilectomía, adenoidectomía, hemorroidectomía y herniorrafia inguinal fue dos o tres veces superior en Canadá que en Inglaterra y Gales. También señala que, en las personas de edad avanzada, la mortalidad provocada por enfermedades de la vesícula biliar resultó dos veces más alta en Canadá que en Inglaterra y Gales, y que la tasa de defunciones por colecistectomía fue cinco veces mayor. Vayda se interroga si esos datos se corresponden con la prevalencia de la enfermedad, con la calidad de las fuentes de información, o con distintas formas de tratamiento y de indicaciones quirúrgicas, y llega a la conclusión que los principales determinantes son las diferencias de organización y pago de los servicios de salud, la “habilitación limitada” para ejercer determinadas tareas y el número de cirujanos y camas del hospital disponibles^51^. 

En 1976, Iván Illich, jesuita, filósofo, teólogo e historiador, publica Némesis médica^79^ allí sostiene que la medicina institucionalizada representa una grave amenaza para la salud de las personas, y considera que sus efectos han alcanzado un nivel epidémico. El libro utiliza fuentes científicas de revistas de gran prestigio, y las obras de Dubos^80^, Cochrane^81^, McLachlan^82^, y McKeown^83^, entre otras. Illich crítica la medicalización en la vida cotidiana, en los presupuestos públicos, el rol perjudicial de la industria farmacéutica, el imperialismo del diagnóstico, los estigmas preventivistas, las ceremonias terminales relacionadas con la muerte, así como la invención y eliminación de enfermedades, llevando su pensamiento a formas radicales al expresar que “la atención médica causa más enfermedades que las que cura”^26^.

La cesárea se convirtió en otra práctica médica controversial. En Brasil se encontró un aumento de las cesáreas en los nacimientos que pasó de un 15%, en 1970, a un 30%, en 1980^84^. Otro estudio de 1985, también de Brasil, halló que a pesar de igualarse la remuneración a los obstetras entre el parto por vía vaginal y por cesárea, el número de cesáreas continuaba aumentando, sobre todo en pacientes particulares, afectando más a sectores sociales de mayores ingresos^85^. Un estudio publicado en 1999 en el *British Medical Journal* calculó que más de 850.000 cesáreas innecesarias eran realizadas cada año en los países de América Latina y el Caribe, usando como medida esperable una tasa del 15%, y observó una correlación positiva y significativa entre el producto nacional bruto per cápita y la tasa de cesáreas, además de tasas más altas en los hospitales privados que en los públicos^86^.

En el año 1991, trabajando en la gerencia de prestaciones médicas del Programa de Atención Médica Integral (PAMI) del Instituto Nacional de Servicios Sociales para Jubilados y Pensionados, Leonardo Werthein me encomendó analizar la mortalidad de los afiliados. Para ello, limitamos el trabajo a la ciudad de Buenos Aires y como *proxy* de mortalidad tomamos el pago de sepelios. Al concluirlo, decidimos comparar los resultados con los muertos totales de la Ciudad de Buenos Aires. La sorpresa fue que el número de muertos del PAMI en la Ciudad de Buenos Aires era mayor que el número total de muertos de la Ciudad de Buenos Aires, cuando por lógica debiera ser menor ya que los muertos del PAMI, residentes en la ciudad de Buenos Aires, constituían una subpoblación de los muertos de la Ciudad de Buenos Aires. Entonces propusimos que a partir de ese momento todo pedido de servicio de sepelio fuera acompañado por el Informe Estadístico de Defunción (IED). A la semana siguiente la Cámara Argentina de Sepelios había conseguido una reunión con los directivos del PAMI, a la cual fuimos invitados para exponer nuestras ideas epidemiológicas. Dejamos a criterio de quienes leen el devenir de este encuentro.

En 2020, Patricia Rosemberg publicó un artículo, a partir de datos del Informe Estadístico de Nacido Vivo, de la Dirección de Estadísticas e Información de Salud del Ministerio de Salud de la Nación de Argentina, para la Ciudad Autónoma de Buenos Aires en el período 2004-2013, analizó la distribución temporal de los días de la semana en que ocurrían nacimientos, encontrando que los nacimientos disminuían significativamente los sábados y domingos en los establecimientos públicos y privados, con una mayor la disminución en los establecimientos privados. Los nacimientos en la semana 37 fueron más frecuentes y tienden a disminuir en los días no laborables. Otro hallazgo fue que, a mayor nivel de instrucción materna, menores fueron los nacimientos durante los fines de semana^87^. Similares resultados se encuentran en otra publicación en Brasil^88^. En Chile, en 2022, en una encuesta realizada a 210 estudiantes universitarias mujeres, un 80% refirió desconocer la menor morbilidad infantil asociada al parto vaginal; más de la mitad consideró la cesárea como una ventaja ya que evitaba el dolor; y un alto porcentaje desconocía los riesgos médicos asociados que conllevan las cesáreas^89^.

A fines de 2022, Jorge Ayala Streck, en su tesis titulada “El sistema de salud público de la Ciudad de Buenos Aires: un análisis desde la epidemiología de servicios y sistemas de salud entre los años 1980 y 2018”^90^, se formula la siguiente pregunta: ¿cuál es el comportamiento en el tiempo de los indicadores seleccionados de epidemiología, servicio de salud, personal y recursos económicos del sistema de salud pública de la Ciudad de Buenos Aires entre los años 1980 y 2018? Para responder a este interrogante, se utilizaron datos oficiales de la Ciudad de Buenos Aires^91^^,^^92^^,^^93^^,^^94^^)^ y se trabajó con cuatro grandes grupos de indicadores: de epidemiología, de servicios de salud, de personal y de recursos económicos. Se analizó la evolución de cada uno de esos grupos y sus interrelaciones y cuando no se encontró la serie completa de datos, el trabajo se limitó a los años en los que había registros. En relación con los valores económicos se trabajó con valores deflactados al año 2019 por el Índice de Precio Implícito del INDEC^90^. Es muy difícil encontrar en la literatura, al menos en Latinoamérica, trabajos con datos oficiales que conformen una serie histórica de casi 40 años como este trabajo, ello lo convierte en una excelente evidencia de lo que venimos postulando, motivo por el cual nos detendremos en los principales resultados^90^. Ayala Streck analizó 38 años de la jurisdicción con más recursos económicos, con personal muy capacitado, con la mayor complejidad tecnológica del país y centro de derivaciones a nivel nacional. La conclusión principal del trabajo es que -como se puede ver a continuación- los indicadores analizados no guardan relación entre sí y que es menester revisar los modos de pensar la administración de los servicios de salud^90^:

1. El total de defunciones, entre 1998 y 2018, por principales causas encuentra un máximo de 34.407 muertes en el año 1998, un mínimo de 27.771 en el año 2018, y una media de 31.005 muertes.
2. Para los valores de tasas de mortalidad bruta del total de defunciones por principales causas entre 1998 y 2018, un valor máximo de 11,30% en el año 1998, un mínimo de 9,05% en el año 2018, una media de 10,12%.
3. Tanto la totalidad de consultas externas como aquellas discriminadas por grupo de especialidad, disminuyen entre 2005-2017. Hay un máximo de consultas externas total de 9.379.171 en el año 2011, un mínimo de 8.095.950 consultas en el año 2017 y una media de 9.005.063. El servicio más demandado fue el de urgencias con 34,5% del total de consultas, y uno de los menos demandados fue el de médico de cabecera con el 1,9% de las consultas registradas.
4. El total de egresos hospitalarios tiene el valor mínimo de la serie en 1990 con 141.708 egresos, alcanza un máximo de 196.975 en el año 2004, y a partir de allí comienza a descender.
5. El total de empleados del Gobierno de la Ciudad Autónoma de Buenos Aires (GCABA) tiene una tendencia creciente a lo largo de la serie estudiada con un máximo de 214.429 empleados en el año 2017, y un mínimo de 127.750 empleados en el año 2002 y una media de 172.639. El número de médicos creció un 7,6 % entre 2014 y 2017, y el de residentes, de todas las especialidades, un 46% entre 2002 y 2017.
6. El total del gasto ejecutado por el Ministerio de Salud del GCABA entre 2000 y 20017, tuvo un máximo en el año 2006 de $ 67.229.193.560, un mínimo de $ 43.301.398.752 en el 2000, y una media de $ 59.342.960.774.
7. El total del gasto ejecutado en los hospitales, entre 2005 y 2017, muestra un máximo de $ 55.322.571.785 en el 2013, un mínimo de $ 47.875.183.259 en el 2005, y una media de $ 52.036.695.767.
8. El gasto ejecutado de personal encontró un máximo del $43.135.509.595 en el 2013, un mínimo del $ 32.980.726.125 en el 2005, y una media de $ 39.784.315.944.
9. El gasto ejecutado en bienes de consumo encontró un máximo de $ 6.811.751.529 en el 2005, un mínimo de $ 4.496.307.260 en el 2008, y una media de $ 5.359.859.496.
10. El gasto ejecutado por servicios no personales encontró un máximo del $ 5.683.888.709 en el 2006, un mínimo de $ 4.185.353.112 en el 2009, y una media de $ 4.757.708.129.
11. En cuanto a los gastos de bienes de uso ejecutado en los hospitales entre 2005 y 2017 arrojó los siguientes resultados: valor máximo de $ 2.693.394.498 en el 2013, mínimo de $ 1.452.212 en el 2006, y una media de $ 1.691.529.410.

Se podrán argumentar muchas explicaciones sobre esos resultados como, por ejemplo, malos sistemas de información, poco compromiso de los profesionales en la producción del dato primario, falta de cultura estadística y epidemiológica, falta de comunicación entre sectores del mismo estado municipal, y todas ellas muy probablemente sean válidas, como venimos viendo en diferentes citas bibliográficas. Pero la pregunta sigue siendo ¿es esto un problema?, ¿para quién?, ¿dónde se discute?, ¿qué opinan las autoridades políticas y técnicas?, ¿qué opinan los legisladores de la Ciudad Autónoma de Buenos Aires?, ¿qué opinan los gremios de los trabajadores y de los profesionales?, ¿qué opinan los habitantes de la Ciudad Autónoma de Buenos Aires?

 En el año 2013 se publicó en el *Journal Primary Care & Community Health* los resultados de una convocatoria a la presentación de artículos sobre *Managerial Epidemiology*, una expresión similar a la epidemiología de los servicios y sistemas de salud*.* En el editorial, que lleva el mismo título de la convocatoria, James E. Rohrer, editor fundador de la revista, y miembro de la Clínica Mayo de Rochester, EEUU, expresa:

*La epidemiología gerencial es un campo que aún tiene que florecer. El tema se convirtió en un tema obligatorio en los programas de posgrado en administración de atención médica hace muchos años, se escribieron libros de texto y se presentaron conferencias en reuniones nacionales. Académicos y legisladores se entusiasmaron con los beneficios potenciales de aplicar los principios y métodos del campo al sistema de atención médica. A pesar de la fanfarria, el campo experimentó un fracaso en el lanzamiento. No se formó ninguna organización nacional o sociedad profesional para promover la epidemiología gerencial. Pocas personas se identificaron como epidemiólogos gerenciales. El término nunca se utilizó como palabra clave, por lo que las búsquedas de artículos arrojan muy poca información. ¿Cómo podría un campo con tanta promesa experimentar casi ningún crecimiento y desarrollo? […] El idealismo de la reforma permeó la formulación conceptual de la epidemiología gerencial […] Los esfuerzos por aplicar la epidemiología a la gestión de la calidad parecen haber sido suplantados por técnicas de ingeniería industrial. Al final, los gerentes vieron poca relevancia en la epidemiología […] A pesar de las repetidas oleadas de reforma de la atención médica, el sistema de atención médica en los Estados Unidos nunca ha pasado de tratar a los pacientes y prevenir enfermedades en las poblaciones. Tal vez sea hora de que nos deshagamos de los viejos supuestos de la epidemiología gerencial. ¿Qué pasará con el campo si se basa en la epidemiología clínica en lugar de la epidemiología de la población? […] Se podría argumentar que la mayoría de los estudios que prueban diferentes enfoques de tratamiento se basan en la epidemiología clínica […] El propósito de esta edición especial de Journal of Primary Care & Community Health es promover el campo de la epidemiología gerencial. Muy pocos de los autores que escribieron los artículos incluidos aquí, habrían descripto su trabajo como epidemiología gerencial o a ellos mismos como epidemiólogos gerenciales.*^95^

En una carta al editor, publicada en ese número del *Journal of Primary Care & Community Health*, se puede leer:

*Hace más de una década, cuando me disponía a escribir un texto sobre epidemiología gerencial, le pregunté a un muy respetado director ejecutivo de un hospital si pensaba que la epidemiología era importante para los gerentes de hospitales; su respuesta fue “¡tenemos una enfermera que hace eso!” […] No estoy seguro de que el valor de la epidemiología como un conjunto de herramientas haya mejorado la toma de decisiones entre los ejecutivos de atención médica en la última década, y esto a pesar del requisito de que la epidemiología se incorpore en los planes de estudios de las maestrías en Administración de Salud […] Las herramientas de la epidemiología brindan información crítica para gerentes y planificadores que buscan predecir la demanda futura de servicios.*^96^

Estas dos últimas citas avalan la universalidad y actualidad del problema que planteamos y la necesidad de la epidemiología de los servicios y sistemas de salud, lo que nos permite plantear que el problema no es desconocido, sino que es ignorado, que no es lo mismo.

En el año 2022, desde el Instituto de Salud Colectiva nos preguntamos sobra la dimensión de las muertes por iatrogenia médica, tomando como un *proxy* para investigarlas los registros de las estadísticas oficiales de mortalidad de Argentina que constituyen las bases de datos de la Dirección de Estadísticas e Información de Salud del Ministerio de Salud de la Nación entre los años 1997 y 2020. En estos registros, la causa básica de muerte se encuentra codificada según la Clasificación Estadística Internacional de Enfermedades y Problemas Relacionados con la Salud (CIE-10) de la Organización Mundial de Salud^97^. En esa clasificación hay dos capítulos que se relacionan con nuestro propósito. El capítulo XXI que lleva el título “Factores que influyen en el estado de salud y contacto con los servicios de salud” (Z00-Z99), pero que no registra muertes ya que los códigos de ese capítulo no pueden ser utilizados para codificar causa básica de muerte. Y el capítulo XX que lleva como título “Causas externas de morbilidad y de mortalidad (V01-Y98)”, del cual tomamos el grupo “Complicaciones de la atención médica y quirúrgica (Y40-Y84)”, y, con especial consideración, los códigos pertenecientes al grupo “Procedimientos quirúrgicos y otros procedimientos médicos como la causa de reacción anormal del paciente o de complicación posterior, sin mención de incidente en el momento de efectuar el procedimiento (Y83 -Y84)”, y también el código el código Y88 “Secuelas con atención médica y quirúrgica como causa externa”, del grupo “Secuelas de causas externas de morbilidad y de mortalidad (Y85-Y89)”.

Los resultados (Tabla 1) suman un total de 21.984 muertes entre los años 1997 y 2020, con un promedio de 912 muertes anuales, con valores máximos acumulados de 20.181 en el código Y83 “Cirugías y otros procedimientos médicos como causa de reacción anormal del paciente o complicación posterior, sin mención de incidente en el momento de efectuar el procedimiento” con el 92,17% de los casos, el segundo código en frecuencia fue el Y84 “Otros procedimientos médicos como la causa de reacción anormal del paciente o de complicación posterior, sin mención de incidente en el momento de efectuar el procedimiento” con un acumulado de 544 muertes.

Los resultados del análisis nos dejan preguntas para entender si esos datos reflejan problemas de calidad o falta de conocimiento para al completar el informe estadístico de defunción, problemas de codificación al analizar el conjunto de causas reportadas en el informe estadístico de defunción y seleccionar la causa básica, o son eventos que expresan iatrogenia.

**Tabla 1:** Causas de muertes según el capítulo XX de la CIE-10 para los códigos Y40-84 y código 88. Argentina, 1997-2020.

Códigos	Años																								
	1997	1998	1999	2000	2001	2002	2003	2004	2005	2006	2007	2008	2009	2010	2011	2012	2013	2014	2015	2016	2017	2018	2019	2020	Total
Y40	2	1	-	-	-	-	-	-	1	1	-	2	-	-	1	-	-	1	1	2	5	10	1	1	29
Y41	1	-	-	1	-	-	-	-	-	-	1	1	-	-	-	-	1	-	-	-	1	-	-	1	7
Y42	-	-	2	-	3	1	-	1	-	-	-	5	-	2	2	3	4	-	-	2	3	1	3	-	35
Y43	5	1	1	1	1	1	-	-	1	4	2	-	4	1	2	-	-	2	1	8	2	2	3	1	43
Y44	15	4	2	7	2	5	3	6	2	2	5	1	2	1	1		1	2	4	1	3	1	2	1	73
Y45	1	-	3	-	-	3	2	5	4	7	1	1	-	2	-	1	-	-	1	7	3	5	6	2	54
Y46	-	-	-	1	-	-	-	-	-	-	-	-	-	1	-	-	-	-	-	-	1	-	-	-	4
Y47	-	3	-	1	-	-	-	-	-	2	2	-	-	-	-	2	-	-	-	2	-	-	1	-	13
Y48	2	-	2	-	-	2	-	1	1	-	-	-	-	-	-	-	1	-	-	3	-	2	2	-	19
Y49	3	4	3	5	-	-	-	1	-	1	-	1	1	1	-	-	1	-	2	-	1	-	-	-	24
Y50	3	5	2	-	-	-	-	-	-	-	-	1	-	1	-	-	-	1	-	5	-	-	1	-	19
Y51	-	1	-	-	-	2	-	-	1	-	-	-	-	-	-	-	-	-	-	-	1	-	-	-	5
Y52	-	3	1	-	2	2	-	-	-	-	1	-	-	1	-	-	-	-	1	1	-	2	-	2	16
Y53	-	1	-	-	-	1	-	-	-	-	-	-	-	-	-	-	-	-	1	1	-	1	1	-	6
Y54	-	-	-	-	1	-	-	-	1	-	-	2	-	-	-	-	-	-	-	-	-	-	-	1	5
Y55	-	-	-	-	-	-	-	1	-	-	1	1	-	-	-	-	1	-	-	-	1	-	-	-	5
Y56	-	-	-	1	-	1	1		1	-	-	-	-	-	-	-	1	-	-	-	-	-	-	-	5
Y57	7	7	5	12	7	1	7	5	6	7	5	7	6	1	6	4	4	1	4	9	5	6	6	2	130
Y58	-	-	1	-	-	1	-	-	-	-	-	-	-	-	-	-	-	-	-	-	-		-	-	2
Y59	8	5	5	3	3	5	1	2	2	2	1	3	6	5	3	4	5	1	2	2	7	9	8	2	94
Y60	6	1	5	1	4	1	-	-	2	-	2	5	1	-	1	2	5	5	7	7	1	5	1	3	65
Y61	1	-	-	-	-	-	1	-	-	1	-	-	-	-	1	1	-	-	-	1	-	-	-	-	6
Y62	-	-	-	-	1	-	-	-	1	1	-	1	-	1	-	-	-	-	-	-	-	-	-	-	5
Y63	-	-	1	1	-	-	-	-	-	-	-	-	-	-	-	1	-	-	-	-	-	-	-	-	3
Y64	-	3	-	-	-	-	1	-	-	-	-	-	-	-	-	-	-	-	-	-	-	-	-	-	4
Y65	4	-	1	1	-	-	2	2	2	3	2	2	2	1	1	1	6	1	1	2	4	-	2	2	42
Y69	2	4	2	1	1	3	1	2			1	2	2	1		2	1	1	1	1	1	3	1	1	34
Y70	-	2		1	2	-	-	-	1	4	2	1	1	-	1	-	-	-	-	4	3	4	3	-	29
Y71	2	9	4	5	7	2	3	3	1	9	1	6	3	4	7	4	4	6	2	9	5	10	9	8	123
Y72	-	-	-	-	-	-	-	-	-	2	-	-	-	-	-	-	-	-	-	-	-	-	-	-	2
Y73	-	-	-	1	-	-	2	-	-	1	-	2	-	-	-	2	2	-	1	2	-	2	-	-	15
Y74	1	1	1	1	-	-	-	-	-	1	-	1	-	1	-	1	-	-	-	-	-	2	1	-	11
Y75	1	-	-	-	1	-	-	-	-	-	1	-	-	-	-	-	-	-	-	-	-	1	1	-	5
Y76	-	-	-	-	-	1	-	-	-	-	-	-	-	-	-	-	-	-	1	-	-	-	-	-	2
Y79	3	10	6	12	12	8	16	17	5	5	4	3	6	8	3	3	2	10	14	15	4	3	1	4	174
Y80	-	-	-	-	-	-	-	-	-	-	-	1	-	1	-	-	-	-	1	-	-	-	-	1	4
Y81	1	-	2	-	-	1	1	-	3	-	-	1	-	1	-	-		-	1	1	-	4	2	1	19
Y82	-	-	-	2	2	-	-	-	7	-	1	1	2	3	7	4	4	2	2	4	-	2	2	-	45
Y83	678	643	586	606	671	653	648	866	987	1.075	1.089	1.084	858	873	1.026	965	1.016	807	940	956	877	935	776	566	20.181
Y84	9	6	13	12	2	5	4	10	8	13	16	15	10	27	30	18	26	19	33	47	65	45	54	57	544
Y88	3	-	2	6	11	9	1	2	3	4	4	3	2	7	5	7	1	1	2	3	-	4	2	1	83
Total	758	714	650	682	733	708	694	924	1.040	1.145	1.142	1.154	906	947	1.097	1.025	1.086	863	1.023	1.095	993	1.059	889	657	21.984

Fuente: Elaboración propia en base a datos del Ministerio de Salud de la Nación. Dirección de Estadísticas e Información de Salud.

### ¿Medicamentos o mercancías?

En EEUU, hasta el siglo XIX, los medicamentos se conseguían de manera libre. Los fabricantes de panaceas, las compañías fabricantes de medicina, autodenominadas “patentes”, y los médicos que preparaban sus propios medicamentos competían entre sí, configurando un mercado caracterizado por medicamentos baratos y consultas médicas muy caras^26^. La AMA se opuso a los medicamentos de patentes, y luchó por tener el control de la información farmacéutica, y así poder regular la medicina de patentes, que según la AMA era realizada por charlatanes y descarados^9^^,^^26^.

En las estrategias de venta de la época, las compañías de fabricantes de medicamentos fomentaron los principios de la moral victoriana basados en el recato de las mujeres ante la posibilidad del examen físico al consultar a los médicos. Así esas compañías invitaban a las mujeres a desistir de ese encuentro y seguir sus indicaciones, que a veces eran cartas con recomendaciones personales enviadas por la propia compañía^26^. En paralelo, la AMA aconsejaba a la población consultar a los médicos, pero sin mucho éxito. Esa situación llevó a la AMA a recopilar todas las maniobras de ventas de la medicina de patente y de los charlatanes, como así los llamaban, y las publicaron con el título *The great American fraud*^98^, de gran repercusión en la sociedad. Todo ello llevó a que, en 1906, el Congreso de EEUU aprobara la ley de “Fármacos y Alimentos Puros”, que inició la regulación federal de los medicamentos^26^. 

Eran tiempos en que la AMA se oponía a todo tipo de publicidad de medicamentos, y por lo tanto solicitó a los dueños de los periódicos que sacrificaran un porcentaje de su tasa de ganancia en función de la salud pública, propuesta que los dueños de los diarios aceptaron. Además varios estados respaldaron esas acciones y promulgaron leyes prohibiendo la publicidad de los medicamentos^26^. La voz de la AMA en tanto voz de la medicina científica se volvía potente, y la siguiente conquista fueron los alimentos para niños fabricados por Nestlé. En una defensa corporativa del conocimiento médico, la AMA planteó que “el conocimiento debe emanar de la profesión médica, no de empresarios capitalistas”^26^ y así se aseguraron la intermediación y los beneficios económicos de tal tarea. La AMA no tardó mucho en conseguir que se dispusieran que las ventas de medicamentos fueran solo bajo receta médica, lo que obligaba a los pacientes a realizar la consulta médica respectiva; del mismo modo que pasó a cobrar la publicidad en sus publicaciones, la cual se convirtió en su principal fuente de ingresos^26^.

La aprobación en 1965, del *Medicare* y *Medicaid* en EEUU, tornó la atención médica mucho más lucrativa, dado su financiamiento público, lo cual terminó por reformular los vínculos entre el personal médico, los hospitales, las escuelas de medicina, las empresas de seguros, la industria farmacéutica, la industria de tecnologías biomédicas y otros negocios ligados al campo de la salud^26^.

Luego del control del mercado, la AMA se propuso instalar en el imaginario social que los medicamentos eran productos inocuos fundamentados en la ciencia. Esa idea se fue desdibujando en el tiempo y alcanzó su mayor expresión en lo que Starr llamó el fin del mandato^26^. A partir de allí se suceden publicaciones críticas, que comienzan a señalar el negocio de la industria farmacéutica, y cómo no escatiman esfuerzos para incrementar la tasa de ganancia, aunque ello signifique disponer en el mercado medicamentos innecesarios o nocivos para la salud de las personas.

Son muchos los libros que desde la segunda mitad del siglo XX denuncian el negocio de la gran industria farmacéutica, y el impacto en la salud de las personas y en el gasto en salud^42^^,^^43^^,^^44^^,^^45^^,^^46^. Silverman y Lee, en una clásica obra de la década de 1970, marcan un camino ideal para el medicamento, conformado por las siguientes etapas: su descubrimiento por parte de los investigadores, su producción por la industria, su prescripción por los profesionales, y el uso correcto por los pacientes. También señalan que ese camino no se cumple casi nunca, y que es excepcional alcanzar esa linealidad, ya que muchas veces el que los produce, el que los prescribe, o quienes lo utilizan, en función de sus intereses o interpretaciones, desvirtúan esa linealidad. Ello significa no solo gastos millonarios inútiles, sino internaciones innecesarias y pérdidas de vidas^40^.

Marcia Angell siempre tuvo un tenor muy crítico en sus trabajos, tanto sobre el sistema de salud de EEUU, como sobre el tema de los medicamentos, críticas que vehiculizó no solo en publicaciones científicas^99^^,^^100^^,^^101^^,^^102^, sino también en la prensa en general^103^^,^^104^. Siempre denunció los fuertes conflictos de intereses en prácticamente todos los campos de la medicina, particularmente, en medicamentos y tecnologías. Así llegó a afirmar que ya no es posible creer en gran parte de la investigación clínica que se publica, confiar en el juicio de médicos de confianza o en directrices médicas autorizadas.

Ella reconoce que no le complacían las conclusiones a las que había llegado durante dos décadas como editora del *New England Journal of Medicine.* Con posterioridad a dejar la dirección de la revista, vuelca su experiencia como editora de esa revista en el libro *La verdad acerca de la industria farmacéutica: cómo nos engaña y qué hacer al respecto*^34^, publicado en 2004*.* En ese libro hace una pormenorizada y fundamentada crítica sobre las grandes empresas de la industria farmacéutica:

*De 1960 a 1980 las ventas de medicamentos bajo receta se mantuvieron más o menos estables en términos del porcentaje del producto interno bruto de los Estados Unidos, pero de 1980 a 2000 se triplicaron. En los últimos años han aumentado a más de doscientos mil millones al año. Por lo demás, desde comienzos de 1980, la industria se ha posicionado como la más lucrativa de los Estados Unidos, y tiene gran ventaja sobre las que le siguen (sólo en 2003 bajó de esa posición al tercer lugar entre las 47 industrias mencionadas en Fortune 500). Entre los múltiples sucesos que contribuyeron a su repentina buena suerte, ninguno tuvo que ver con la calidad de las drogas que vendían las empresas.*^34^

En la parte final del libro hay un párrafo cuya validez excede a los EEUU y tiene cada vez mayor vigencia.

*Los medicamentos por prescripción son una parte fundamental de la atención médica. Los estadounidenses necesitan fármacos nuevos que sean buenos y tengan precios razonables. Sin embargo, la industria farmacéutica no satisface esa necesidad. Hay una enorme distancia entre su retórica y sus prácticas. Impulsadas por su afán de lucro, parece que se esfuerza cada vez más en alcanzar la autodestrucción. Su manera actual de hacer negocios no es sostenible. Tanto el gobierno federal, como la profesión médica han sido cooptados por la riqueza y el poder de las grandes farmacéuticas, pero tarde o temprano todo esto tendrá que cambiar. El subsidio a los medicamentos por prescripción de Medicare le dará a la industria un gran empuje, pero no durará mucho. Los que pagan los medicamentos (el gobierno, las aseguradoras y los individuos) sencillamente no tienen dinero para seguir manteniendo la industria en su forma actual. Y el público está enojado.*^34^

En su libro no podían faltar los visitadores médicos que son quienes explican los beneficios de los medicamentos a los profesionales, y que representan, en un alto porcentaje, la única fuente de actualización para los profesionales. Marcia Angell describe el trabajo de los visitadores médicos en EEUU de la siguiente manera:

*…en 2001 la industria empleó 88 mil representantes de ventas para que visitaran a los médicos en sus consultorios y en los hospitales con el propósito de promocionar sus productos. Ello viene a ser uno por cada cinco o seis médicos en actividad, sin contar internos y residentes. Estos representantes o visitadores médicos, como se les denomina, se encuentran por todos lados en el mundo de la medicina. Por lo general, son jóvenes, atractivos y muy amables. Caminan por los pasillos de casi todos los hospitales importantes del país, al acecho de la oportunidad de hablar con el equipo médico, abriéndose camino con obsequios (tales como libros, pelotas de golf, raquetas para eventos deportivos). En muchos hospitales de enseñanza médica, los visitadores organizan almuerzos para internos y residentes y, entre tanto, les hablan sobre sus medicamentos. Estas reuniones de “comida, halagos y amistad”, como las llaman, crean un sentido de reciprocidad en los médicos jóvenes, que tienen largas vidas de prescripciones por delante. […]. A los visitadores se les permite asistir a conferencias médicas, pueden ser invitados a los quirófanos y salas de curaciones, y a veces están presentes cuando los médicos examinan a los pacientes en clínicas o en la cama de hospital. A menudo, se les deja creer a los pacientes que los visitadores son médicos, una creencia que se refuerza cuando los visitadores ofrecen consejos sobre el tratamiento. Las exigencias de la atención a los asegurados ocupan cada vez una mayor parte del tiempo de los médicos y, por lo tanto, los visitadores médicos tienen cada vez más dificultades para encontrarse personalmente con ellos. Ya casi no los atienden o, si lo hacen, reducen sus visitas a pocos minutos: “Deje el paquete en la puerta”. La intensa competencia por el tiempo y la atención de los médicos ha impulsado la aparición de nuevas empresas que se especializan en entrenar a los visitadores médicos en discursos rápidos y eficaces.*^34^

En 2013, Peter Gøtzsche publicó Medicamentos que matan y crimen organizado: Cómo las grandes farmacéuticas han corrompido el sistema de salud^19^. Este libro junto al de Marcia Angell^34^, publicados con nueve años de diferencia entre sí, constituyen dos obras icónicas sobre el accionar de las grandes compañías farmacéuticas. La edición en español del libro tiene la presentación de Joan Laporta, profesor de Terapéutica y Farmacología Clínica de la Universitat Autónoma de Barcelona y, para la edición en inglés, de Richard Smith exdirector del British Medical Journal. En sus primeras hojas hay comentarios que avalan fuertemente los contenidos del libro como los de Drummond Rennie, vicedirector del JAMA (Journal of the American Medical Association) y de Henry H. Bauer, editor emérito del Journal of Scientific Exploration.

En la presentación del libro, Joan Laporte considera que:

*El título de este libro no es una exageración. Personas que lo han leído han experimentado una ira creciente a medida que avanzaban por la clarividente e implacable descripción del profesor Peter Gøtzsche sobre prácticas reiteradas de la industria farmacéutica: extorsión, ocultamiento de información, fraude sistemático, malversación de fondos, violación de las leyes, obstrucción a la justicia, obstrucción a la aplicación de la ley, falsificación de testimonios, compra de profesionales sanitarios (alquiler, dicen los cínicos), manipulación y distorsión de los resultados de la investigación, alienación del pensamiento médico y de la práctica de la medicina, divulgación de falsos mitos en los medios de comunicación, soborno de políticos y funcionarios, y corrupción de la administración del Estado y de los sistemas de salud. El resultado: centenares de miles de muertes cada año, atribuibles a los efectos adversos de unos medicamentos que no era necesario tomar y al despilfarro de recursos públicos (públicos por ahora, en España). Uso este lenguaje fuerte porque este libro cuenta cosas fuertes, que deben ser conocidas. Además, las documenta con precisión.*^19^

*Furia basada en pruebas* es el título de la presentación de Smith, una muy buena descripción para un libro que presenta las pruebas con una pasión acorde a la gravedad de lo que describe^19^. *Smith* resalta que la postura de Gøtzsche se centra en los fallos del sistema farmacológico, relacionados con el descubrimiento, fabricación, comercialización y regulación de los fármacos, lo cual no significa que él niegue la importancia de los medicamentos, y para no dejarlo solo a Gøtzsche con sus críticas, recupera una frase de William Osler, uno de los cuatro profesores que fundaron *Johns Hopkins Hospital,* “sería bueno para la humanidad, y malo para los peces, que lanzáramos al mar todos los medicamentos”. Gøtzsche también aporta en el libro otra frase de Osler “el ansia de tomar medicamentos es quizá la gran diferencia entre los humanos y los animales”. Ambas frases de Osler han perdido vigencia, ya que en estas épocas los animales consumen más antibióticos que los humanos, y en algunos países la diferencia es del 80%^105^. Esa situación ha aumentado la resistencia de las bacterias a los antibióticos y agrava el problema de las infecciones intrahospitalarias.

Gøtzsche denuncia que “en los países ricos las enfermedades causadas por medicamentos son ya la tercera causa de muerte, detrás del infarto y el cáncer. En los países menos ricos, ni se sabe”^19^. En el libro se señalan faltas éticas, adulteración de resultados en ensayos clínicos y escritores fantasmas, en una conjugación de intereses, en los que intervienen alianzas entre las agencias de regulación de fármacos, los comités examinadores de guías clínicas y de listas de medicamentos, el marketing farmacéutico, los médicos y sus asociaciones, los enfermos y sus asociaciones, las revistas especializadas y los periodistas^19^.

En 2018, la *Cochrane Colaboration* apartó de su cargo a Gøtzsche, que los denunció por la creciente falta de colaboración democrática y pluralismo científico. Su expulsión se realizó por seis votos a favor y cinco en contra^106^. Archibald Cochrane hubiese renunciado a la fundación que lleva su nombre de haber estado vivo, no solo por acordar con las ideas de Gøtzsche, sino por ver cómo sus ideales fueron traicionados y mercantilizados.

Al analizar el devenir de los medicamentos desde fines del siglo XIX, con los fabricantes de panaceas, las compañías fabricantes de medicina de patentes y los médicos que preparaban sus propios medicamentos^26^ -que condujeron a la AMA a publicar *The great American fraud*^98^*-* y compararlo con las graves denuncias que contienen los libros de Angell^34^ y Gøtzsche^19^ en los inicios del siglo XX, es indudable que resulta difícil visualizar un camino lineal de progreso científico, más bien cabe señalar una circularidad borgiana, producto de intereses de actores que expresan un profundo desprecio por la vida y la salud de las personas.

### De lo que dicen los pacientes, a lo que dicen las máquinas

Durante los últimos cinco siglos se incorporaron a las prácticas profesionales del campo de la salud diferentes tecnologías que impactaron en el encuentro entre el profesional y el paciente, e incidieron en la progresiva transformación de médicos generales en médicos especialistas. Las tecnologías cambiaron el trabajo médico, reduciendo la autonomía profesional y socavando el carácter artesanal de su trabajo. Todo ello en pos de una falsa objetividad que con el tiempo pasó a conocerse como “medicina basada en la evidencia”^19^.

Esos profesionales, seducidos por las tecnologías y las técnicas, subvaloraron las subjetividades de quienes acudían a sus consultas. La tecnologización de los procesos de trabajo profundizó el distanciamiento de la medicina con el humanismo, lo cual trajo graves consecuencias en los trabajadores de la salud, expresados a nivel personal en *burnout,* desmoralización y desmotivación y, a nivel institucional, en fragmentación y desafiliación. Todo ello evidencia cómo los trabajadores de la salud fueron también víctimas del éxito de las tecnologías. Ello se hizo muy evidente en la pospandemia, cuando los trabajadores manifestaron su descontento por cómo vivenciaban su trabajo, produciéndose desafiliaciones masivas que no hicieron más que representar una fractura en la cultura de esas instituciones y en las representaciones sociales sobre la medicina existentes en la sociedad^107^.

#### Los descubrimientos generaron nuevos problemas

Durante el siglo XVI, las lentes convexas permitieron el surgimiento del microscopio, que produjo grandes avances al poder descubrir y describir los microbios que hasta entonces eran invisibles a la vista humana^9^. También en ese siglo, la química comenzó a ser utilizada para diagnósticos a través de la visualización de la orina. A partir de allí se inició su progresiva utilización para diagnosticar enfermedades. A comienzos del siglo XX, en EEUU comienzan a surgir laboratorios comerciales que ofrecían servicios de exámenes químicos y microscópicos, pero es recién con la Primera Guerra Mundial que muchos médicos por primera vez pasan por la experiencia de trabajar con laboratorios de diagnósticos^9^.

En el siglo XVII, la medicina utilizó la palabra y la vista para realizar diagnósticos, y en el siglo XVIII se sumaron técnicas manuales para el examen físico que, complementadas con la disección de cadáveres, fueron utilizadas para descifrar los enigmas de la enfermedad a través de la anatomía, la fisiología y la patología. En el siglo XIX, ocurren varios descubrimientos significativos, como el estetoscopio por Laennec, en 1816; el oftalmoscopio por Hermann von Helmholz, en 1851; el laringoscopio por Philipp Bozzini, en 1855; y los rayos X por Wilhelm Röntgen, en 1895; entre otros^9^^,^^55^^,^^108^. En general, esos descubrimientos fueron presentados con potencialidades exageradas, y no se tardó en reconocer la carga subjetiva de quienes lo utilizaban, es decir, los observadores no eran neutrales y sus intereses no siempre reflejaban cientificidad^9^.

En esos años, la biología, la fisiología y la bacteriología pasaron del nivel orgánico al celular, consolidándose la dimensión científica de la medicina a través de procesos de cuantificación. Esos procesos sustentaron criterios de normalidad, que permitieron clasificar a las personas^9^^,^^26^^,^^109^. La cuantificación pasó a dominar la medicina, creando un ideal falso, que Stephen Gould calificó como “la falsa medida del hombre”^110^. Así los valores de laboratorio que definen enfermedades fueron cambiando en el tiempo y esos cambios construyeron más diabéticos o hipertensos, que comenzaron a utilizar medicamentos y consultar especialistas. Los cambios de los valores de referencia significan grandes ganancias para la industria de la enfermedad^19^.

El teléfono fue otro descubrimiento que se incorporó en la práctica médica. En California, hay registros de publicidades en 1878, que invitan a los pacientes a realizar consultas telefónicas las 24 horas^9^. En la Segunda Guerra Mundial se hicieron pruebas de enviar electrocardiogramas por cables telefónicos.

El dominio de la concepción mecánica del ser humano^111^, y la idea de que la enfermedad residía en los órganos, favoreció la especialización de los médicos que fueron abandonando la medicina general seducidos por el arsenal tecnológico. El conocimiento y manejo de las tecnologías era fundamental para transformarse en especialista. En EEUU, en la segunda mitad del siglo XIX, los médicos especialistas tenían mala reputación, pero en 1929, casi uno de cada cuatro médicos privados era especialista, y en 1969 la proporción aumentó a tres de cada cuatro^9^^,^^26^.

En las primeras décadas del siglo XX, las prácticas de la medicina privada comenzaron a organizarse en grupos de profesionales, que fueron criticadas por

*…convertir la medicina, de una profesión en un negocio. Los contadores y las sistematizadas disposiciones financieras revelaban un espíritu comercial rara vez visto en la práctica individual. El organizado procedimiento de pacientes por médicos y máquinas pareció similar a los métodos de la industria de producción en masa. La división del paciente entre los especialistas del grupo -medicina de “tienda de departamentos”- producía una atmósfera impersonal que desagradaba a los pacientes [...] faltaba el importante rasgo del contacto personal entre médico y paciente, con la debida integración y estimación del valor de los detalles.*^9^

La implementación de diferentes tecnologías en el campo de la salud no tardó en demostrar las consecuencias no racionales de su uso. En 1930, se encontró que el 90% de los electrocardiogramas no beneficiaban ni al paciente, ni al médico y entre mediados de 1950 y la década de 1960, diferentes hospitales de EEUU informaron que el número de pruebas de laboratorio se duplicaba cada cinco años, sin corresponderse con un aumento de pacientes. En la década de 1970, el número de pruebas de laboratorio en EEUU pasó de 2.000 millones en 1971 a 4.500 millones en 1976. La automatización de los laboratorios fomentó e hizo posible la conformación de paquetes fijos de pedidos de análisis para determinadas patologías, como parte de los estudios de rutina^9^. Los gastos en EEUU en pruebas de laboratorio pasaron, entre 1974 y 1976, de 8 mil millones a 12 mil millones^9^. Con el uso de los rayos X la situación fue similar: entre 1938 y 1958 se encontró que en seis diagnósticos se había sextuplicado su uso^9^.

Tinsley Harrison, autor de *Harrison´s principles of internal medicine*, en 1944 describía el proceso de atención de la siguiente manera:

*…la actual tendencia hacia un historial de cinco minutos, seguido por una verdadera batería de pruebas especiales que duran cinco días, con la esperanza de que el conejo del diagnóstico surja súbitamente del sombrero del laboratorio.*^9^

En 1975, el presidente de la AMA, David Rogers, señaló en un discurso:

*Conforme nuestras intervenciones se han vuelto más minuciosas, también se han vuelto más caras y riesgosas. Hoy, de este modo, no es insólito encontrar a un frágil anciano que entra en el hospital (y queda) ligeramente confuso, deshidratado y un tanto peor al tercer día de su internación, porque sus primeras 48 horas en el hospital las pasó sometiéndose a una serie abrumadora de exhaustivos estudios diagnósticos en varios laboratorios o en la sala de radiología.*^9^

Es indudable que las tecnologías ampliaron las capacidades diagnósticas de la medicina, pero en ese proceso, la observación de los signos y síntomas por parte del médico y la escucha del relato del paciente fueron siendo anuladas ante la presunta objetividad científica que brindaban las tecnologías. Con todo ello, el paciente pasó a quedarse callado y quieto^112^.

#### Tecnologías para la fábrica

Al incorporarse las tecnologías en el campo de la salud recrean el modelo fabril bajo la dirección del *homo sapiens*, planteando la toma de decisiones respaldada en números y planes que se proyectan al futuro. Esos cantos de sirenas sedujeron y seducen a los profesionales de la salud, pero sus prácticas cotidianas también le señalan lo finito de esa ilusión y la complejidad del juego, y en esas encrucijadas sus cuerpos crujen y sus corporeidades sufren.

En 1970, en EEUU, surgió el concepto de “medicina defensiva” como una acción de protección de los profesionales ante la industria del juicio, situación que se propagó más adelante por las Américas. Por ejemplo, la American College of Surgeons, en 1972, encontró que, en sus casi 16.000 miembros, más de la mitad de los pedidos de estudios complementarios se realizaban para protegerse de futuros juicios^9^.

Las tecnologías siguieron su avance y los nuevos descubrimientos trajeron el ecógrafo, la tomografía, la resonancia, etc., todas ellas se sumaron a las instituciones, en general, sin ningún otro propósito que aumentar la rentabilidad, ya que la evaluación ética y de efectividad no existían y, si se realizaban, era con posterioridad a su implementación^12^. En la actualidad, la incorporación de sofisticadas tecnologías y robots avanzan en los hospitales, y sus costos son muy significativos. Por ejemplo, en Argentina, un tomógrafo puede costar 1 millón de dólares, un resonador de última generación puede llegar a los 2 millones de dólares, y un robot Da Vinci para cirugías tiene un precio de entre 1,5 y 2 millones de dólares, con un costo anual de mantenimiento de 140 mil dólares. Luego de la compra de los equipamientos, se los incorpora al patrimonio como bienes de capital y deben ser amortizados, y esa amortización, en general, no se relaciona con el juramento hipocrático.

El optimismo con las tecnologías era tal que, en 1956, con las primeras computadoras, un grupo de biomédicos imaginó un futuro en que:

*La sala de espera del futuro servirá a ocho médicos; será completamente a prueba de sonido. El mobiliario consistirá en sillas de contorno, que darán un suave masaje. Una televisión en colores deleitará la vista, y una suave música permitirá relajar toda angustia… El interrogatorio para la historia clínica será indoloro. Mediante una grabación se hará un grupo de preguntas de preferencia, y con las respuestas se perforará una tarjeta. Nada quedará al azar, y mediante cibernética, la tarjeta pronto quedará depositada en una ranura que ofrecerá los tres diagnósticos más probables. Luego, en un piso móvil, el paciente será transportado al laboratorio. Todos los procedimientos de laboratorio serán hechos a máquina, un solo cerebro electrónico que extraerá sangre hará todo el análisis, incluido el químico… La información se presentará en (otra) tarjeta nuevamente con los tres diagnósticos más probables.*^9^

Ese optimismo del grupo de biomédicos se contradice con las condiciones de trabajo de la mayoría de los trabajadores de salud en las Américas. También podemos afirmar que poco y nada ha cambiado en cuanto al uso irracional de las tecnologías: hoy asistimos a consultas a través de Whatsapp o Zoom, y a estudios en los que es la máquina la que le indica al paciente lo que tiene que ir haciendo durante el procedimiento.

En el año 2020, Noelia Cassani Laham, analizó los avisos publicitarios de la revista *Alta* de Aerolíneas Argentinas, durante el período 2013-2017. El análisis se basó en aspectos estéticos, comunicacionales, semiológicos, médicos y sanitarios. Una de las conclusiones fue “que el 69% de las publicidades implicaban procesos de medicalización” resultado que se alcanzó luego de analizar junto a distintos especialistas las diferentes tecnologías, técnicas, medicamentos o métodos ofrecidos, observándose además que “en la mayoría de los casos la efectividad era dudosa o nula, e incluso en algunos casos el riesgo al cual se exponía al paciente era entre moderado o alto”^113^.

La rutinización y automatización en el uso de las tecnologías, la omnipotencia de la razón, la instrumentalización del conocimiento y la deshumanización en los procesos de cuidado y atención de las personas, determinaron la profunda crisis que atraviesa el campo de la salud, desde hace décadas y a nivel mundial. Crisis que afecta lo humanitario, lo económico y lo vocacional, tanto en jóvenes egresados, como en el conjunto de los trabajadores^114^.

## A MANERA DE CIERRE

Hay una situación recurrente en la agenda de los gobiernos de distintos países y de distintas ideologías y es que los objetivos de atención a la enfermedad son más relevantes que la salud entendida como un derecho social que no es sinónimo de atención médica. Lo paradojal de esa situación es que la mayoría de las enfermedades tienen fuertes relaciones con situaciones de privación social ligadas a la alimentación, la vivienda, el medio ambiente y el trabajo, es decir, tienen una determinación social. Por ello sostenemos que se necesita más salud, no más medicina, y que esas acciones de salud se relacionan con el cumplimiento de los Derechos Económicos, Sociales y Culturales^115^, los cuales se deben traducir en acciones políticas y no limitarse a acciones científicas que responsabilicen a las víctimas, o se limiten a su descripción. Toda esa discusión permanece oculta por la fuerte medicalización de la sociedad^116^.

La epidemiología de los servicios y sistemas de salud es una acción muy necesaria como propuesta técnica para abordar los problemas señalados, pero su acción es insuficiente desligada de acciones y decisiones políticas que pongan en discusión y publicicen la autoridad cultural de la medicina científica.

Sería muy necio, y está muy alejado de nuestro propósito el negar la importancia de los descubrimientos y alcances de la ciencia en muchos aspectos de los procesos de salud-enfermedad-atención-cuidado. Lo que si proponemos es publicizar las situaciones descriptas en relación con la medicina científica, entendiendo por ello la construcción espacios públicos que retiren los problemas de la esfera privada y los coloquen en el espacio de lo público, en todas aquellas situaciones que se lucra con el sufrimiento y el dolor de las personas, ya sea a través de prácticas o prescripciones innecesarias, como el inventar enfermedades, prescribir medicamentos o estudios innecesarios^19^^,^^21^^,^^117^.

Es indudable que la medicina científica produjo una revolución cultural a partir del siglo XX, por la cual logró apropiarse del problema de la enfermedad y convertirse en la autoridad cultural del campo de las prácticas de los procesos de salud-enfermedad-atención-cuidado^12^. Por todo ello, limitar la interpretación de este ensayo a la propuesta de la epidemiología de los servicios y sistemas de salud significaría reducir el problema a una cuestión técnica, y ocultar que estamos frente a un problema público de estructura sociocultural^12^.

Es muy necesaria la problematización y la publicización de la medicina científica como autoridad cultural de los procesos de salud-enfermedad-atención-cuidado, la cual es utilizada para demostrar desinterés y así ocultar los verdaderos intereses. La problematización debe centrarse en acciones dirigidas a debatir en el espacio social y la agenda pública las graves consecuencias sobre la salud de las personas y los presupuestos públicos de ese interés del desinterés^17^. La publicización debe enfocarse en mantener el problema en la esfera de lo público y que no sea cooptado por expertos, o simplificada por los medios de comunicación social, es decir, negarse a la construcción de autoridades que quiten el problema de la esfera pública, ya que la fuerza de cambio no está en el campo de la salud, sino por fuera de él^118^.

De no problematizarse las dimensiones socioculturales de los procesos de salud-enfermedad-atención-cuidado, se profundizarán realidades sociales conformadas por altos porcentajes de la población sin asistencia sociosanitaria, conviviendo con importantes fracciones de la sociedad que seguirán consumiendo prácticas asistenciales, medicamentos y tecnologías que afectan o afectarán su salud bajo la autoridad sociocultural de la medicina científica.

Colocar en discusión en la agenda pública la autoridad cultural del problema público de la salud no es tarea sencilla, ya que los problemas públicos no pueden desligarse de las experiencias y las perturbaciones que esas mismas experiencias provocan, a nivel individual y colectivo, y que no hacen más que expresar la fuerte influencia de lo cultural en la conformación del complejo problema de los procesos de salud-enfermedad-atención-cuidado^17^. 

Las trabajadoras y los trabajadores de la salud, profesionales y no profesionales, han visto en las últimas décadas deteriorarse sus condiciones materiales y simbólicas de trabajo, produciéndose desafiliaciones institucionales y rupturas de vínculos con las comunicades y los equipos. Todo ese proceso no fue gratuito para la salud de muchas de estas personas, que siguen sin entender el juego que juegan. ¿No será el momento de comenzar a sacarnos de encima esa pesada mochila de la autoridad cultural y construir nuevos vínculos con las personas, los colectivos sociales y las organizaciones en sus territorios? ¿No están pagando esas trabajadoras y esos trabajadores un precio demasiado alto por jugar un juego que no solo no los favorece sino que los perjudica?
